# Autologous Millifat Grafting as a Reconstructive Strategy for Complex Lower Limb Defects in a Diabetic Patient After Necrotizing Fasciitis

**DOI:** 10.7759/cureus.101252

**Published:** 2026-01-10

**Authors:** Karen Rodríguez Franco, Juan Darío Alviar Rueda, Maria Camila Vega Corredor, Camila Castillo Hernandez

**Affiliations:** 1 Plastic and Reconstructive Surgery, Hospital Universitario de Santander, Bucaramanga, COL; 2 Plastic and Reconstructive Surgery, Hospital Internacional de Colombia, Bucaramanga, COL

**Keywords:** adipose tissue/transplantation, autologous fat grafting, fasciitis, leg ulcer, limb salvage, necrotizing

## Abstract

Lower limb ulcers, especially in patients with diabetes mellitus, represent a major therapeutic challenge, particularly when complicated by severe infections such as necrotizing fasciitis. These conditions often require extensive surgical interventions, including amputation. We present the case of a 68-year-old female patient with poorly controlled type 2 diabetes mellitus who developed extensive necrotizing fasciitis in the left lower limb following trauma with plant material, resulting in a large soft tissue defect. The patient was not a candidate for regional or distant flaps due to her non-revascularizable micro- and macroangiopathic disease. A prior CT angiography showed complete occlusion of the anterior and posterior tibial arteries, evaluated by Orthopedics, who indicated amputation of the limb. Due to the patient’s refusal to undergo amputation, autologous millifat grafts were selected for the salvage treatment strategy. This case highlights the potential of this technique as a limb salvage tool, demonstrating its effectiveness in complex clinical scenarios by promoting tissue regeneration and avoiding major ablative procedures.

## Introduction

Lower limb ulcers are a significant health concern, particularly in patients with peripheral vascular diseases, diabetes mellitus, or chronic venous insufficiency [1]. When these ulcers become complicated by bacterial infections, the severity of the condition increases considerably [2]. In some cases, the infection may progress to necrotizing fasciitis, a rare but potentially life-threatening condition that causes rapid destruction of soft tissues and often requires aggressive surgical intervention to avoid ablative management [3].

The treatment of ulcers complicated by superinfection presents a therapeutic challenge, as conventional strategies do not always achieve complete or rapid healing, thereby endangering the viability of the affected limb [4]. In this context, the use of autologous fat tissue has emerged as a promising therapeutic alternative. This technique not only supports tissue regeneration but may also enhance vascularization, paracrine function, and the microenvironment of coverage defects, promoting the healing of chronic and difficult-to-treat wounds [5].

This article discusses the use of millifat-type fat grafting in the treatment of coverage defects secondary to necrotizing fasciitis. A biopsy was performed for definitive diagnosis through histopathology and culture. Before surgical debridement, the patient underwent CT angiography, which showed gas in the tissues and thickening of the fascia, as well as obstructive involvement of the anterior and posterior tibial arteries. Therefore, the orthopedic service recommended amputation of the leg, representing a clinical case of a diabetic patient. The benefits of this technique are explored in terms of limb preservation, its ability to accelerate healing time, and its potential to reduce the need for major surgical interventions such as amputation.

## Case presentation

A 68-year-old female patient with a history of poorly controlled type 2 diabetes mellitus (HbA1c 12.3) presented with a clinical course of one and a half months, secondary to blunt trauma with vegetal material (wood), initially resulting in excoriation on the anterior aspect of the left leg. This lesion progressed to edema, erythema, warmth, pain, formation of blisters on the dorsum of the foot, and semicircumferential necrosis of the left leg (approximately 70% of the circumference), extending toward the dorsum of the foot and the second and third toe phalanges.

There was exposure of the extensor tendons of the foot and toes, as well as bone exposure of the distal tibia and fibula, with involvement of the small saphenous vein and sural nerve, after the first surgical debridement with the removal of the hard eschar that had been present for a month and a half. A biopsy was performed for a definitive diagnosis of necrotizing fasciitis through histopathology and culture. Before surgical debridement, the patient underwent CT angiography, which showed gas in the tissues and thickening of the fascia, as well as obstructive involvement of the anterior and posterior tibial arteries. Therefore, the orthopedic service recommended amputation of the leg.

Initial management included broad-spectrum antibiotic therapy (linezolid - vancomycin), along with sequential surgical debridements and negative pressure wound therapy for 22 days. However, osteotendinous exposure persisted.

Adequate surgical debridement is a necessary first step to initiate proper granulation; however, it is not sufficient to generate it in areas with exposed structures such as tendons, neurovascular structures, and bones with significant loss of important tissue. The use of flaps was also not an option due to the poor vascular substrate.

**Figure 1:**
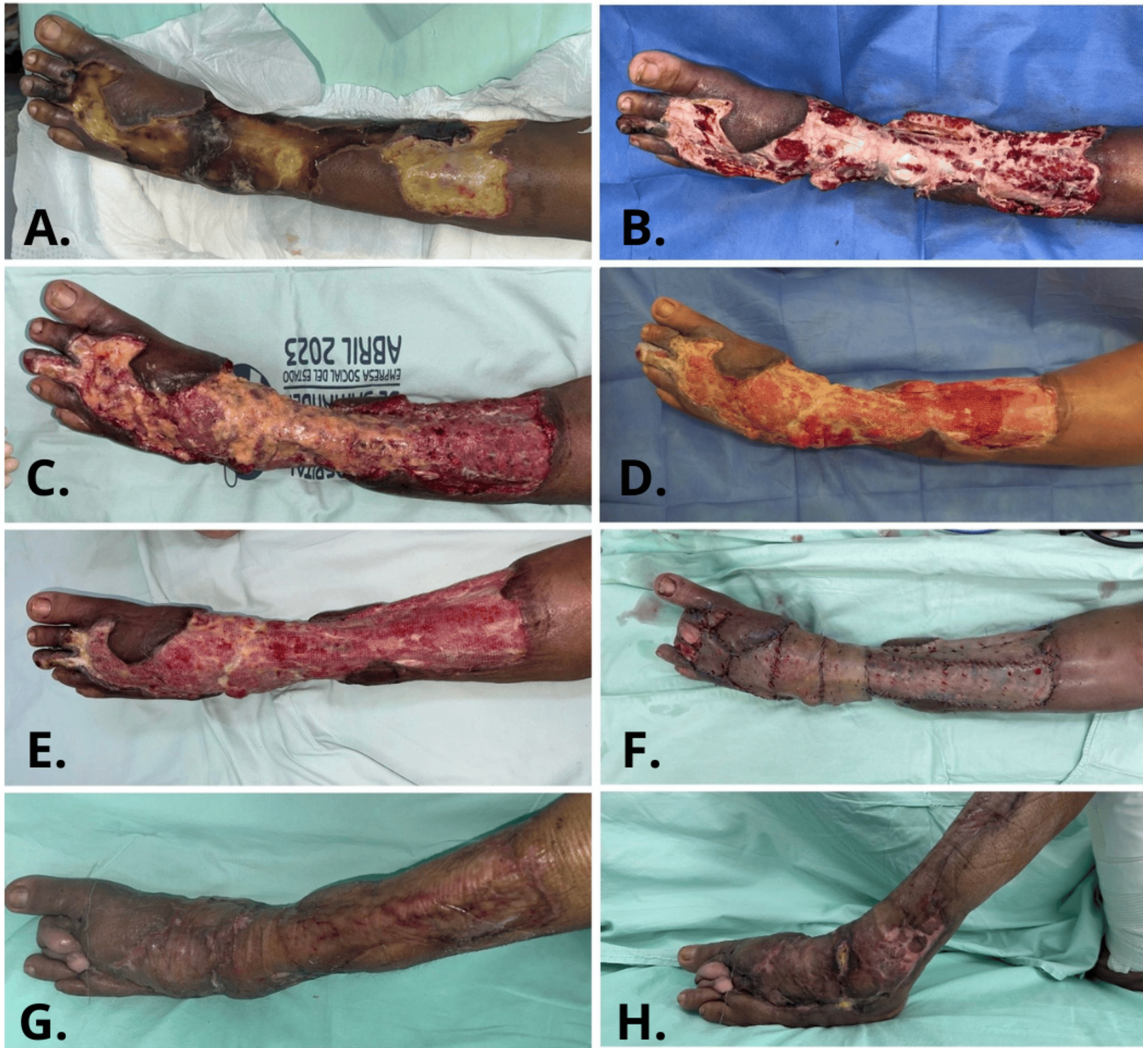
A. Preoperative view. B. After surgical washing and debridement of the limb with osteomyo­tendinous exposure. C. Immediate postoperative appearance after autologous millifat fatgraft. D. First dressing change (postoperative day 3). E. Fourth dressing change (postoperative day 9). F. Intraoperative view of partial-thickness skin graft placement. G and H. Satisfactory evolution of the skin graft.

After ruling out local infection, the patient met the inclusion criteria for the autologous millifat grafting protocol. Satisfactory outcomes were observed, with appropriate granulation tissue formation by postoperative day 10, at which point definitive coverage was achieved with partial-thickness skin grafts (Figure 1).

The patient's functional status was assessed four months postoperatively using a series of tests measuring strength, balance, flexibility, and lower extremity mobility such as the straight leg raise, single-leg squat, and sit-to-stand test.

## Discussion

Lower limb ulcers affect between 0.6% and 3% of patients over the age of 60, and increase to more than 5% in those over 80 years old. Their prevalence varies between 1.9% and 13.1% across different populations [1].

The morbidity burden of this condition stems from its multifactorial pathophysiology, involving neuropathy, vasculopathy, immunopathy, mechanical stress, and deformity [2]. These factors drastically increase the risk of hospitalization, functional loss, emotional and financial detriment to the patient. Hard-to-heal wounds significantly raise the cost of treatment. A venous leg ulcer (VLU) can cost a person in the United States up to $19,000 USD per year due to recurrent treatments, while a diabetic foot ulcer (DFU) can lead to amputation, with an estimated cost between $38,000 and $54,000 USD [6].

Diabetes-related foot ulcers (DFU) requiring amputation carry a five-year mortality risk higher than some oncologic diseases. A 30.5% mortality rate is reported for DFU compared to 9.0% for breast cancer and 80.0% for lung cancer [7].

Based on the foregoing, there is a clear need to effectively address these types of ulcers through reconstructive surgery to reduce hospitalization time and the risk of amputation.

In terms of chronic wound treatment, several approaches exist, including a variety of dressings such as films, hydrocolloids, foams, hydrogels, alginates, and hydrofibers, as well as skin substitutes developed through tissue engineering, growth factors, negative pressure therapy, and hyperbaric oxygen. However, these methods do not always demonstrate complete effectiveness [8].

Surgical debridement plays a crucial role, especially in the context of ulcers complicated by infection or necrotizing fasciitis, which often require several reconstructive procedures.

Methods such as skin grafts or flaps are used to cover defects involving substantial tissue loss. However, in some cases, such procedures are not feasible due to comorbidities, the patient’s functional status, or exposure of neurovascular or osteotendinous structures. In this case, the use of skin grafts alone was also not viable due to the exposure of vital structures that required a granulation bed, which was provided thanks to the use of fat grafts.

In recent years, there has been growing interest in the application of cellular therapies for wound management. In this regard, autologous fat grafting has been proposed as a treatment option for these complex cases [9].

The use of autologous adipose tissue is an emerging therapeutic option, mainly employed in cases where the wound bed is not yet suitable for skin grafting or where there are exposed specialized structures. Initially described as a cosmetic procedure within aesthetic plastic surgery, its scope of use has since expanded.

Recently, therapy involving adipose-derived stem cells (ADSCs) has gained popularity due to their pluripotency, self-renewal capability, and ability to stimulate the secretion of regenerative cytokines. These cells are believed to promote wound healing through a paracrine mechanism [10], modulating macrophages, T cells, and B cells to reduce inflammation, secreting vascular endothelial growth factor (VEGF) to facilitate angiogenesis, and promoting fibroblast and keratinocyte proliferation and differentiation [11]. Additionally, they produce anti-fibrotic cytokines and can differentiate into microvascular endothelial cells [12]. Moreover, exosomes contained within ADSCs are key components of the paracrine pathway and play a fundamental role in the effectiveness of stem cell therapy. These secreted organelles, rich in proteins, lipids, nucleic acids, and carbohydrates, are involved in various functions, including extracellular matrix remodeling and molecular signaling. Furthermore, ADSCs are present at approximately 500 times the concentration found in an equivalent amount of bone marrow, and their harvesting involves significantly lower morbidity [12].

Advantages and disadvantages

Among its advantages, this is considered a multifunctional procedure applicable across a wide range of indications, from chronic ulcers and difficult-to-heal wounds to aesthetic and reconstructive applications. Additionally, as autologous fat tissue, there is no risk of rejection or immunologic reaction [3]. The fat graft is harvested through small incisions, which are aesthetically favorable for the patient. Moreover, by using 3 mm liposuction cannulas, less damage is caused to surrounding tissues, resulting in shorter recovery times compared to other surgical procedures, with a significant reduction in hospital stay duration and cost reduction. It has also been shown to improve the texture and appearance of skin in the treated area.

However, the risk of reabsorption must be considered at different rates, which can lead to inconsistent results [13]. The procedure also requires specialized personnel trained in reconstructive plastic surgery.

Surgical technique

Under spinal anesthesia and following aseptic and antiseptic preparation, the surgical team performed irrigation and debridement of a 403 cm² coverage defect on the dorsum of the left foot and semicircumferential region of the left leg due to previously treated necrotizing fasciitis with broad-spectrum antibiotics. The wound bed presented exposed extensor tendons and metatarsals of the first and second toes.

Tumescent infiltration was performed in the hypogastric adipose tissue using a solution of 150 cc of 0.9% normal saline + 0.1 cc of epinephrine + 10 cc of 1% lidocaine, administered with a 3 mm infiltration cannula. Subsequently, liposuction was carried out using a 3 mm grater cannula, yielding 180 cc of fatgraft, which was collected in a hermetically sealed glass reservoir [14].

The harvested fat was allowed to decant for 20 minutes, and the aqueous and blood components were removed. The unfiltered and non-centrifuged fat graft was then applied directly to completely cover the defect in the left lower limb. The area was dressed with a paraffin gauze, sterile plastic dressing, and elastic bandages without compression.

Dressings were changed according to protocol. The first dressing change was performed on the third postoperative day after keeping the leg in a horizontal position for 2 days to prevent loss of the fat graft due to gravity and to promote its integration, supported by a semi-occlusive covering that maintained its adherence while generating angiogenesis, improvement of the microenvironment of the defect, the anti-inflammatory effect and the multilineage cell differentiation characteristic of the millifat, and subsequently the dressing was uncovered and changed every two days while maintaining the paraffin dressing. By day 9, healthy granulation tissue was observed, and by day 11, there was a 38% reduction in defect size with complete coverage of bone and tendon structures. Partial-thickness skin grafts were applied on postoperative day 14. These outcomes were achieved with a single application of autologous fat grafts.

The use of autologous fat grafts in the treatment of necrotizing fasciitis represents an innovative therapeutic strategy that may significantly improve tissue regeneration, reduce the need for amputations, and support faster functional recovery.

POSAS scale application

Outcomes were assessed using the POSAS (Patient and Observer Scar Assessment Scale) to monitor scar appearance from both the patient’s and the observer’s perspectives, allowing for more objective measurement. This scale includes parameters such as pain, itching, color, texture, harshness, elasticity, and vascularity. Each part of the scale consists of several items scored on a numerical scale from 0 to 10, where 0 generally indicates the best possible condition (no scar or minimal scarring) and 10 represents the worst possible condition (severe or abnormal scarring) [15].

As shown in Tables 1-2, which display the scores assigned by both the observer and the patient before and after surgical management, there is a clear improvement in tissue condition and the healing process.

**Table 1:** Observer component of the Patient and Observer Scar Assessment Scale (POSAS)

Aspect	Pre-Surgical	Post-Surgical	Delta Change
Vascularity	10	3	7
Pigmentation	9	3	6
Tissues elevation	9	3	6
Rugosity	10	3	7
Flexibility	10	3	7
Surface Area	10	3	7
General opinion	10	3	7

**Table 2:** Patient component of the Patient and Observer Scar Assessment Scale (POSAS)

Aspect	Pre-Surgical	Post-Surgical	Delta Change
Pain	10	2	8
Itch	8	2	6
Color	9	4	5
Harshness	9	4	5
Tissues elevation	10	2	8
Rugosity	9	3	6
General opinion	9	2	7

The interpretation of the POSAS scale in this clinical case shows significant improvement in scar evolution from both the patient's and the observer's perspectives. Before the intervention, the general opinion score given by the patient was 9, while the observer rated it 10, indicating a clinically severe scar with a negative impact on the patient's well-being and quality of life. After the intervention, the patient's general score decreased to 2, indicating a considerable reduction in subjective symptoms such as pain. The observer rated the scar at 3, reflecting notable improvements in clinical features such as vascularity, pigmentation, and flexibility.

These changes in scores demonstrate the effectiveness of the intervention, with statistically significant responses, showing improvement in both the subjective perception of the patient and the objective assessment by the healthcare provider, indicating a successful recovery and a functionally and aesthetically less severe scar.

## Conclusions

The use of autologous fat grafting represents an innovative and effective therapeutic alternative in the management of complex wounds and coverage defects in the lower limbs, particularly in patients with comorbidities such as diabetes mellitus and vascular insufficiency, in whom conventional healing is often impaired. Fat grafts enhance tissue regeneration and angiogenesis, attributable to the presence of adipose-derived stem cells, which contribute to significant improvements in vascularization and the healing of chronic wounds, thereby reducing the risk of severe complications such as amputation. Moreover, the technique is minimally invasive and associated with lower morbidity compared to other procedures for improving wound beds to achieve definitive coverage with partial-thickness skin grafts. Additionally, it reduces the risk of infection, improves the quality of the grafted skin, and facilitates faster recovery, resulting in shorter hospital stays. Limb preservation is the product of a collaborative effort of specialties such as infectious diseases, plastic surgery, physical therapy, endocrinology, and physiatry. This includes infection control, limitation of micro- and macroangiopathy, ischemia, neuropathy, and hyperglycemia, ultimately providing the reconstructive option that offers coverage and final healing to the affected limb.

The clinical case presented demonstrates that the application of fat grafting can be an effective strategy included in the limb salvage process, achieving adequate coverage of exposed structures and improving the functionality of the affected limb. However, further clinical studies and long-term follow-up are needed to validate the effectiveness and optimize the technique of fat grafting in patients with complex ulcers and necrotizing fasciitis. Standardized protocols must be developed to define their role within reconstructive strategies in plastic and reconstructive surgery.
